# Antimicrobial stewardship, therapeutic drug monitoring and infection management in the ICU: results from the international A- TEAMICU survey

**DOI:** 10.1186/s13613-021-00917-2

**Published:** 2021-08-26

**Authors:** Christian Lanckohr, Christian Boeing, Jan J. De Waele, Dylan W. de Lange, Jeroen Schouten, Menno Prins, Maarten Nijsten, Pedro Povoa, Andrew Conway Morris, Hendrik Bracht

**Affiliations:** 1 Antibiotic Stewardship Team, Department of Hygiene, University Hospital Münster, Munster, Germany; 2 Department of Critical Care Medicine, Ghent University Hospital, Ghent, Belgium; 3 Department of Intensive Care Medicine, Universitair Medisch Centrum, University Utrecht, Utrecht, The Netherlands; 4 Department of Intensive Care, Radboud UMC, Nijmegen, The Netherlands; 5 Dept. Biomedical Engineering, Eindhoven University of Technology, Eindhoven, The Netherlands; 6 Department of Intensive Care Medicine, University of Groningen, Groningen, The Netherlands; 7 Polyvalent Intensive Care Unit, Hospital de São Francisco Xavier, Lisbon, Portugal; 8 Division of Anaesthesia, University of Cambridge, Cambridge, UK; 9 Clinic for Anesthesiology, University Hospital Ulm, Ulm, Germany

**Keywords:** Antimicrobial stewardship, Therapeutic drug monitoring, Critical care, Multiresistant bacteria, Antibiotic

## Abstract

**Background:**

Severe infections and multidrug-resistant pathogens are common in critically ill patients. Antimicrobial stewardship (AMS) and therapeutic drug monitoring (TDM) are contemporary tools to optimize the use of antimicrobials. The A-TEAMICU survey was initiated to gain contemporary insights into dissemination and structure of AMS programs and TDM practices in intensive care units.

**Methods:**

This study involved online survey of members of ESICM and six national professional intensive care societies.

**Results:**

Data of 812 respondents from mostly European high- and middle-income countries were available for analysis. 63% had AMS rounds available in their ICU, where 78% performed rounds weekly or more often. While 82% had local guidelines for treatment of infections, only 70% had cumulative antimicrobial susceptibility reports and 56% monitored the quantity of antimicrobials administered. A restriction of antimicrobials was reported by 62%. TDM of antimicrobial agents was used in 61% of ICUs, mostly glycopeptides (89%), aminoglycosides (77%), carbapenems (32%), penicillins (30%), azole antifungals (27%), cephalosporins (17%), and linezolid (16%). 76% of respondents used prolonged/continuous infusion of antimicrobials. The availability of an AMS had a significant association with the use of TDM.

**Conclusions:**

Many respondents of the survey have AMS in their ICUs. TDM of antimicrobials and optimized administration of antibiotics are broadly used among respondents. The availability of antimicrobial susceptibility reports and a surveillance of antimicrobial use should be actively sought by intensivists where unavailable. Results of this survey may inform further research and educational activities.

**Supplementary Information:**

The online version contains supplementary material available at 10.1186/s13613-021-00917-2.

## Background

Antimicrobial resistance (AMR) is a concern in many regions of the world [1]. Antimicrobial use and horizontal spread of bacteria have been recognized as the most important drivers of resistance and its dissemination [2, 3]. In addition to infection prevention measures, antimicrobial stewardship (AMS) is an essential step to optimize consumption of antimicrobials and thus reduce bacterial resistance [4–6].

Intensive care units (ICUs) are burdened by large numbers of patients with infections and sepsis, high antimicrobial use, and high rates of resistance [7]. Therefore, the rational management of anti-infectives and infection control measures are core competencies of intensive care medicine specialists [8]. In 2016, experts from the European Society of Intensive Care Medicine (ESICM) and the European Society of Clinical Microbiology and Infectious Diseases (ESCMID) in collaboration with the World Alliance Against Antimicrobial Resistance (WAAAR) held a round table meeting on antimicrobial resistance [9]. Besides the improvement of awareness for AMR and surveillance, the engagement of intensivists in multidisciplinary AMS teams in the hospital was recommended. Along the same lines, AMR and AMS were identified as integral components of the intensive care medicine research agenda to be addressed in the future [10].

In addition to a reduction of antimicrobial use to diminish ecological pressure in the ICU environment, pharmacologic optimization of antimicrobial administration is recognized as an important target in critically ill patients. Standard dosing regimens for many antimicrobials were shown to be associated with extremely variable drug levels [11], potentially causing unacceptable rates of underdosing and adverse outcomes [12, 13]. Furthermore, the international ADMIN-ICU survey found a marked heterogeneity of dosing strategies and the use of therapeutic drug monitoring (TDM) [14].

The “Antimicrobial Stewardship, Therapeutic Drug Monitoring and Early Appropriate infection Management in European ICUs” (A-TEAMICU) survey was initiated by the Infection Section of the ESICM to gain insights into the development of AMS programs and TDM practices in ICUs since the publication of ADMIN-ICU in 2015 and the 2016 round table meeting on antimicrobial resistance. Results from this survey can be the basis for educational initiatives on behalf of ESICM by providing real-world information on the dissemination and structure of AMS and TDM. We hypothesized that AMS programs, the availability of TDM and the use of pharmacologically optimized infusion of antimicrobials have markedly increased in comparison to prior surveys.

## Methods

### Survey population

The survey was endorsed by ESICM and six national professional societies (Australia and New Zealand, Germany, Brazil, United Kingdom, the Netherlands, and Portugal). The societies used their respective members email addresses to send a link to an online survey. Due to data protection regulations, the numbers of professionals who were contacted and their email addresses remained unknown to the A-TEAMICU investigators. The survey was conducted in English, participation was voluntary (i.e., there was no financial remuneration), and respondents remained anonymous. The number of participants from a single center or hospital was not controlled.

### Data collection

The A-TEAMICU study group consists of experts with clinical expertise in intensive care medicine, infectious diseases, and antimicrobial stewardship who are members of the “Infection Section” of ESICM. The idea to conduct this survey was formulated during a section meeting. Participation in this project was open to everyone interested in the topic. Using a recent survey on AMS by the “ESCMID Study Group in Antimicrobial Stewardship (ESGAP)” as a starting point [15], questions were modified to the ICU setting by the authors of this publication. The questions were preformulated and discussed by the group in video/telephone conferences. All questions were consented by the whole group. The final core survey consisted of 23 questions. Dependent on the participants’ answers, 13 additional questions were asked (Additional file 1). The questionnaire was divided into four sections (hospital information, organization of an antimicrobial stewardship program, therapeutic drug monitoring, education in antimicrobial stewardship). The A-TEAMICU survey used the “Survey Monkey” platform which was provided by ESICM.

Before starting the survey, ethical approval was sought at the University of Ulm (Germany). The local ethics committee waived the need for formal ethical approval of A-TEAMICU as an anonymous online survey of clinical practice.

Descriptive statistics were expressed as total numbers and percentages for categorical variables. Sample size calculations were not performed, as it is not possible to estimate the number of participants before the survey. Due to this, we only performed univariate analyses for categorical variables, using the Chi-squared test. A *p*-value < 0.05 was considered statistically significant.

## Results

### Demographic information

**Fig. 1:**
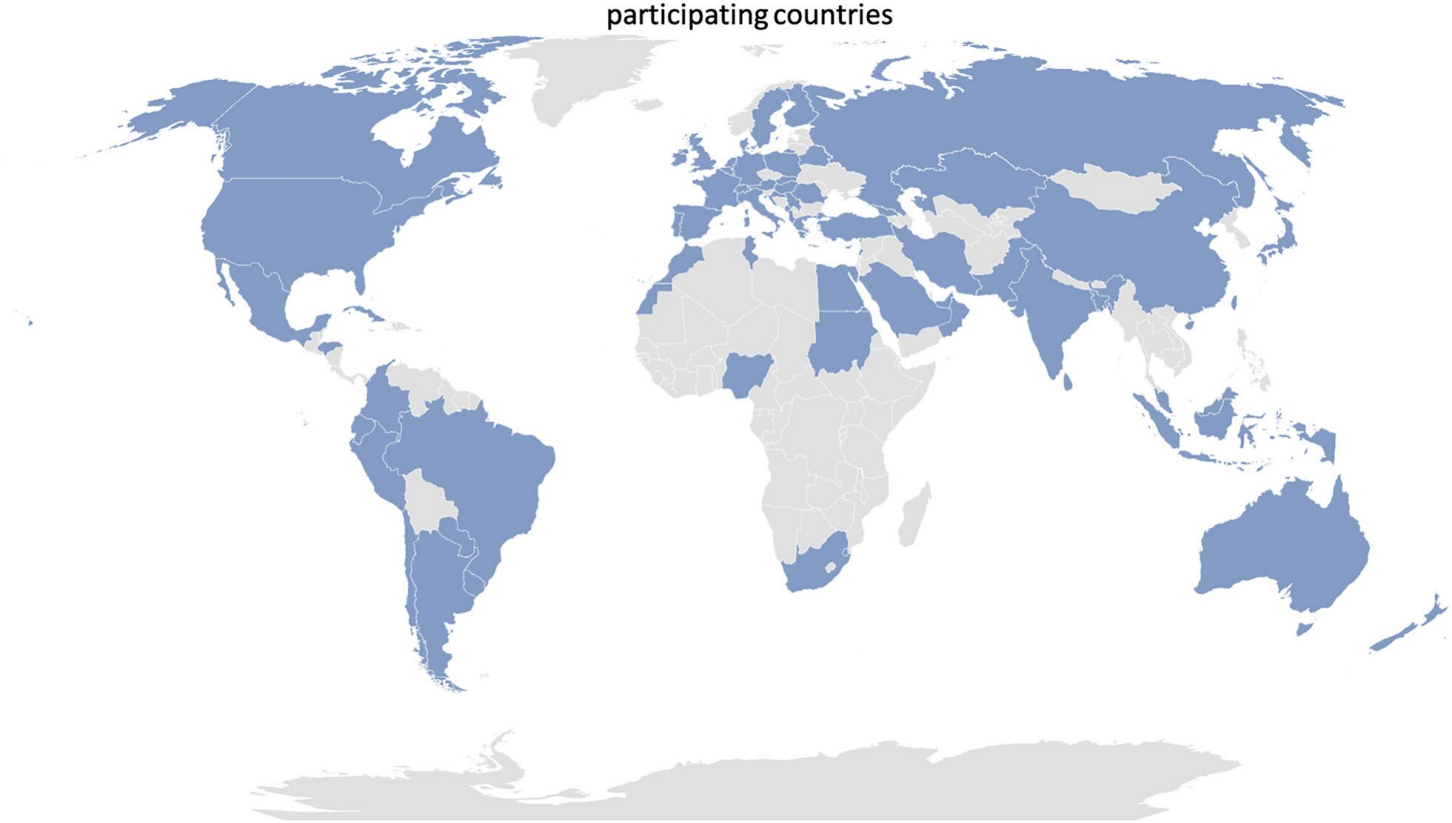
Countries from which participants took part in the A-TEAMICU survey

In total, 812 participants from 71 countries responded to the survey (Fig. 1). The countries of origin were classified using the criteria of the Statistics Division of the United Nations [16] (Table 1). The majority (85%) of respondents worked in high-income countries, while 14% participated from upper-middle and lower–middle-income countries. Seven respondents did not provide information about their country of origin.

**Table 1:** Demographic information

Characteristic	Numbers (%)
Region of origin	
Europe and Central Asia	641 (80%)
Latin America and Caribbean	61 (8%)
East Asia and Pacific Region	38 (5%)
Middle East and North Africa	27 (3%)
South Asia	24 (3%)
North America	8 (1%)
Sub-Saharan Africa	6 (0.7%)
Type of hospital	
Academic hospital	416 (52%)
Non-academic teaching hospital	275 (34%)
General non-teaching hospital	114 (14%)
Numbers of ICU beds	
≤ 10	156 (19%)
11–20	226 (28%)
21–30	123 (15%)
31–40	89 (11%)
41–50	44 (5%)
> 50	163 (20%)

To our knowledge, this is the largest survey on this topic.

### Part 1: hospital information and demographics

Respondents were generally experienced ICU clinicians and a substantial proportion considered themselves unit leaders in infection management. 8% of participants have experience in the ICU of less than 2 years, 15% have 2–5 years, 19% 5–10 years, 33% 10–20 years, and 25% reported experience of more than 20 years. Approximately half reported having received specific training in antimicrobial therapy or infection management. 35% of respondents considered themselves the most qualified intensivist on their respective service regarding infection management. Detailed information about the hospital types and the numbers of ICU beds are included in Table 1.

Infectious disease (ID) specialists were available for consultations in 67% of hospitals, with a further 16% available as external consultants. Clinical microbiologists were available for in-house consultation in 60% of hospitals, while 22% had availability of external consultation. A lack of ID support was reported by 16% of participants; 17% cannot consult a clinical microbiologist. Most respondents use an electronic medical record in the ICU (59%).

### Part 2: AMS and infection management

A formal antimicrobial stewardship program (ASP) existed in 69% of hospitals, and 63% of participants had an ASP available in their ICU. Common members of the A-team were clinical microbiologists (62%), infectious disease specialists (57%), clinical pharmacists (50%), and infection prevention specialists (21%). In 77% of ICUs with availability of an ASP team, the intensivist was member of the team. The A-team visited the ICU weekly in 37% of hospitals; 41% had rounds of the A-team more often (several times a week in 21%, daily in 20% of ICUs). 14% of respondents had the A-team only available on demand.

A restriction of selected antimicrobials with the necessity for formal authorization was in place in 62% of hospitals. Detailed information on the methods used for the implementation of restrictions is provided in Table 2.

**Table 2:** Details on antimicrobial stewardship programs (ASP) and therapeutic drug monitoring (TDM)

Characteristic	Numbers (%)
Infection management and ASP	
ASP available in the ICU	499 (63%)
Any restriction of antimicrobials	478 (62%)
Availability of local treatment guidelines	619 (82%)
Quantitative monitoring of antimicrobial use	409 (56%)
Availability of cumulative antimicrobial susceptibility reports	506 (70%)
Use of therapeutic drug monitoring (TDM)	435 (61%)
Use of prolonged/continuous infusion of antimicrobials	539 (76%)
Implementation of restriction of antimicrobials	
Antibiotic order forms	198 (41%)
Pre-authorization	156 (33%)
Post-authorization	125 (26%)
Post-prescription review	130 (27%)
Telephone feedback	114 (24%)
Formulary restriction	89 (19%)
Mandatory bedside consultation	91 (19%)
Computerized alerts	56 (12%)
Automatic stop orders	43 (9%)
Availability of guidelines/standards	
De-escalation of therapy	410 (50%)
Duration of therapy	377 (46%)
Dose optimization	357 (44%)
Discontinuation	266 (33%)
Antimicrobials with available TDM	
Glycopeptides	387 (89%)
Aminoglycosides	333 (77%)
Carbapenems	138 (32%)
Penicillins	131 (30%)
Azole antifungals	117 (27%)
Cephalosporins	76 (17%)
Linezolid	68 (16%)
Echinocandin antifungals	66 (15%)
Colistin	52 (12%)
Quinolones	44 (10%)
Daptomycin	36 (8%)
Antimicrobials with prolonged/continuous infusion	
Penicillins	410 (76%)
Carbapenems	405 (75%)
Glycopeptides	269 (50%)
Cephalosporins	163 (30%)
Linezolid	71 (13%)
Azole antifungals	39 (7%)

Most respondents (82%) had local guidelines for the treatment of infectious diseases available in their hospitals. In 87% of hospitals with local guidelines, recommendations were based on local susceptibility patterns. Further information on the availability of guidelines/standards is provided in Table 2. Only 19% reported to have no specific guidance documents in the ICU. 52% of participants had a written ICU policy requiring prescribers to document the indication of antimicrobials in the patient records.

The quantity of antimicrobials prescribed was monitored in 56% of ICUs. In these hospitals, daily defined doses (DDDs) were the most used statistical measure (41%), followed by days of therapy (DOTs) in 29%. 26% of participants were unsure of the details of antimicrobial usage surveillance in their hospital.

Regarding cumulative antimicrobial susceptibility reports, only 70% of participants had these data available for their ICUs. 17% were uncertain about their local status, while 13% reported a complete lack of such information.

43% of respondents had a system of mandatory bedside consultation by ID specialists for special types of infections in the ICU. These consultations were designated for endocarditis (64%), invasive fungal infections (61%), *Staphylococcus aureus* bacteremia (55%), prosthetic joint infection (48%), and infection of vascular prostheses (42%).

### Part 3: use of TDM and prolonged/continuous infusion of antimicrobials

75% of participants had written guidelines for antimicrobial dosing in their ICU, where 77% used a local guideline and 23% had national guidelines. Therapeutic drug monitoring of antimicrobial agents was used in 61% of ICUs. Where TDM was available, drug measurements were performed by the clinical chemistry service in 63% of hospitals, by the clinical pharmacy in 16% of cases and by the microbiology department in 11%. Advice on the clinical use of drug measurements was provided by various specialists, including intensivists (72%), microbiologists (30%), ID specialists (29%), clinical pharmacists (28%), and clinical chemistry specialists (14%). Antimicrobials available for TDM are listed in Table 2.

To elucidate whether ASP had an association with the use of TDM, we compared respondents by their ASP status (yes or no, 545 vs. 215, respectively) and their reported use of TDM. In the cohort of respondents with an ASP, 331 used TDM, while 156 did not (58 respondents did not provide answers to both questions). In comparison, in the cohort without availability of an ASP, 95 respondents used TDM, while 91 did not (29 respondents did not provide answers to both questions). In a univariate analysis, the presence of an ASP had a significant association with the use of TDM (odds ratio 2.03 [1.44–2.87], *p* < 0.001).

76% of respondents used prolonged and/or continuous infusion of antimicrobials in their ICU (see Table 2 for list of antimicrobials). 29% of participants had a TDM available for every antimicrobial that they give extendedly. We did not find an association of the availability of an ASP with the use of prolonged or extended infusion of antimicrobials. When focusing on beta-lactams specifically, there was also no significant association.

### Part 4: education

In 53% of ICUs, education on antimicrobial stewardship was provided to physicians. This training was mandatory in 23% of cases, all other ICUs offered education on AMS on a voluntary basis. Common topics discussed included antimicrobial resistance (88%), streamlining and de-escalation (73%) management of specific syndromes (e.g., *S. aureus* bacteremia, pneumonia, endocarditis) (64%), use of TDM (51%), use of restricted agents (50%), oral switch (40%), and results from local audits and prevalence surveys (23%).

## Discussion

This international survey among intensive care specialists adds knowledge to results of previous inquiries that have explored the local organization of AMS in the intensive care setting in Germany [17] and France [18]. To the best of our knowledge, therapeutic drug monitoring in the ICU has only been surveyed once in the past [14] and we are able to provide a current perspective on the evolving use of this technology. The number of participants in A-TEAMICU was considerably larger than in those surveys.

Slightly more than 60% of respondents have an ID specialist and a clinical microbiologist available at their hospitals, while the remainder must rely on external consultations or cannot access this resource at all. This finding reflects both the known shortages of ID physicians and clinical microbiologists and a growing centralization of microbiology services in laboratories detached from hospitals [19]. While it is evident that the core responsibility for the management of infections is in the hands of the intensivist, the option to acquire specialized input should be available, as it is a valuable addition to good patient care [20, 21].

Notwithstanding these infrastructural challenges, the widespread implementation of formal AMS programs in hospitals and ICUs is an encouraging finding, reflecting a growing dedication of the medical community to the prevention of AMR. This is all the more encouraging because of the documented lack of standardization of training in AMS, infectious diseases, and infection prevention in many countries [22]. As recommended by contemporary guidelines [4], clinical microbiologists, infectious disease specialists, and clinical pharmacists are common members of the AMS team in hospitals with AMS programs. Our results are comparable with the findings of a recent survey in four European countries, analyzed AMS on a hospital level, albeit without special focus on the ICU [15]. In the A-TEAMICU cohort, 77% of respondents with an AMS program in their ICU report that the intensivist is a member of the AMS team, demonstrating that intensive care medicine specialists are actively engaging in AMS activities. Furthermore, about half of participants have received specific training in antimicrobial therapy or infection management, adding to the profile of the intensivist as “infection manager.”

The availability of local guidelines in 82% of ICUs is a finding that warrants attention. The adaption of empiric antimicrobial therapy to local epidemiology is essential to guarantee adequacy of therapy and simultaneously curb overtreatment. Thus, every hospital ought to provide such recommendations to their staff. In our survey, 87% of hospitals where such guidelines are available incorporate local resistance data. Again, this leaves room for improvement. On a positive side, a considerable number of ICUs in the A-TEAMICU cohort have special guidelines and recommendation on antimicrobial de-escalation, duration of therapy, TDM, and antimicrobial discontinuation. This finding is encouraging, as it reflects recent developments in the field of intensive care medicine [23, 24]. Regarding surveillance in general, cumulative antimicrobial susceptibility reports for the ICU are only available in 70% of ICUs and only 56% of participants have a monitoring of antimicrobial use. These data are indispensable for both therapeutic decisions and AMS programs in general and intensivists should actively demand the provision of surveillance information.

Therapeutic drug monitoring of anti-infective substances is widely available in ICUs and intensivists are the predominant discipline to advise on the use of TDM in the A-TEAMICU cohort. We found an association of the use of TDM with the availability of an ASP, which is a plausible finding as the pharmacologic optimization of antimicrobial is a central tenet of antimicrobial stewardship. In addition to glycopeptide and aminoglycoside antibiotics, approximately 30% of respondents have the ability to monitor β-lactams. This proportion is higher than expected and demonstrates the growing use of pharmacokinetic optimization of antimicrobial therapy in the ICU. A recent position paper by ESICM (published after A-TEAMICU) explicitly recommends the use of β-lactam TDM in critically ill patients [25] and many ICUs appear to already pursue these goals.

Concurrent with the finding of an increased use of TDM is the extensive use of prolonged and/or continuous infusion of antimicrobials by 76% of respondents. As pharmacologically reasonable, this practice predominantly focuses on β-lactam antibiotics, but 50% also use extended infusion regimens for glycopeptides. The latter result was unexpected, as current guidelines recommend this practice explicitly for patients in whom therapeutic targets of vancomycin are not attained with intermittent bolus dosing [26]. Whether the continuous infusion of vancomycin may also be used to reduce toxicity is still a matter of debate. The widespread use of prolonged/continuous infusion of β-lactams is a surprising development from results of previous surveys, where only 20–30% of participants used this technique [14, 27, 28]. Although our questions did not differentiate between extended and continuous infusion, we found a clear move away from bolus application of time-dependent antibiotics. This likely reflects the growing evidence base for this therapeutic concept [23]. At the same time, recent evidence has identified a need for education on various pharmacologic topics related to the use of antimicrobials [29]. Of note, in our population we did not find a clear association of the use of prolonged/continuous infusions with the presence of an ASP. On a practical level, the extended infusion of suitable antimicrobials is easier to implement than TDM, as the latter has technical requirements beyond the ICU. We speculate that intensivist do not “need” an ASP to introduce prolonged/continuous infusion, whereas the provision of TDM is a more general infrastructural challenge for a hospital. Thus, it might be argued that an ASP not only propagates the use of TDM but also works to provide the possibility to monitor antimicrobial concentrations. This might be an explanation for the influence of an ASP on the use of TDM, without a clear influence on the use of prolonged/continuous infusion.

Taken together, the results of the A-TEAMICU survey provide insight into many aspects of contemporary infection management in the ICU. As the prevalence of infections in critically ill patients remains high [7], knowledge of diagnostics, antimicrobial pharmacology, and infection prevention are essential for the practice of intensive care medicine. Antimicrobial stewardship as a “bundle” of coordinated actions to optimize the use of antimicrobials [30] has established itself in many ICUs and many intensivist are engaged in AMS. Some components of AMS have their primary application in the ICU setting, e.g., the optimization of antibiotic therapy by means of TDM or de-escalation. Thus, intensivists are principle proponents who assume leadership in these topics [10, 12, 31]. Professional organizations, like ESICM, might use results from A-TEAMICU to expand and refine their engagement on AMS and TDM in intensive care medicine. Besides research undertakings, the provision of education on infection management appears to be another relevant field of activity. This might also include the formulation of “best-practice-statements.” A specific example might be the availability guidelines for empirical therapy that must not only be available but also based on local epidemiology. ICUs without such guidelines could use recommendations by specialist organizations to advance this issue with their hospital management. As the management of infections in the ICU needs an interdisciplinary framework to achieve the best possible outcomes, specialist organizations should also assume a leading role in the development of interprofessional cooperation.

A limitation of our survey is a probable selection bias of participants. The invitation to the survey was primarily distributed to ESICM members, and intensivists not affiliated with this society were harder to reach. We tried to reduce this bias by asking several national societies to use their respective members’ addresses to achieve a higher dissemination among the target population. Still, it is unlikely that participants outside of these professional societies took part. Furthermore, respondents with a personal interest in infections and antimicrobials are more likely to accept an invitation to provide information about their current practice. The same probably holds true for intensivists who work in an environment (both ICU and the hospital as a whole) that has an emphasis on infection management and AMS. Therefore, a “positive” selection of hospitals with a good structure and intensivists with a personal dedication to the management of infections cannot be excluded. Additionally, we cannot exclude the possibility that several participants from the same hospital or ICU provided answers. This selection bias might also be applicable to the high rate of TDM-use. Still, we do not consider this possibility disadvantageous, as this might reflect a type of contemporary “best practice.” Lastly, a majority of participants of A-TEAMICU came from high-income and upper middle-income countries, where hospital infrastructure and health system funding can be expected to be better than in lower-income countries. This will have an influence on the availability of staff (e.g., pharmacists, ID specialists) and technology (e.g., TDM, laboratory resources), limiting the global generalizability of the survey results.

A way to reduce potential selection/participation biases in future surveys might be a more stringent control of participants. As an example, allowing only one person per hospital or ICU to provide answers to the survey might diminish the impact of institutions where various aspects infection management are considered essential and are thus endued with sufficient resources. However, this might potentially reduce anonymity and thus either prevent colleagues from participation, or introduce a social desirability bias, where participants provide answers that they consider to be seen as favorable. Besides limitations relevant to participants, the set of questions used in A-TEAMICU was not comprehensive with regard to the detailed execution of AMS in participating hospitals. As an example, we did not assess how feedback on AMS interventions was provided to prescribers. This aspect and other omissions were necessary to limit the size of the questionnaire and the time needed to complete the survey. Still, A-TEAMICU focuses on AMS form a primarily intensive care medicine point-of-view and if a participant feels that AMS is implemented in their ICU, we believe that this is a relevant information. The goal of this survey was not to assess a type of “best practice of AMS” in ICUs but rather gain insight into the dissemination of basic components of ASPs.

In conclusion, many ICU physicians who participated in the A-TEAMICU survey have AMS in their ICUs. A number of “core elements” of AMS are implemented in the respondents’ hospitals (Table 2). Of particular interest, TDM of antimicrobials and optimized administration of antibiotics are broadly used.

## Supplementary Information

**Additional file 1.** A-TEAM-ICU Questionnaire.

## Data Availability

The datasets used and/or analyzed during the current study are available from the corresponding author on reasonable request.

## Abbreviations

AMS: Antimicrobial stewardship
TDM: Therapeutic drug monitoring
A-TEAMICU: Antimicrobial stewardship, therapeutic drug monitoring, and early appropriate infection management in European ICUs
ESICM: European Society of Intensive Care Medicine
ICU: Intensive Care Unit
AMR: Antimicrobial resistance
ESCMID: European Society of Clinical Microbiology and Infectious Diseases
WAAAR: World Alliance Against Antimicrobial Resistance
ESGAP: ESCMID Study Group in Antimicrobial Stewardship
ID: Infectious diseases
ASP: Antimicrobial stewardship program
DDD: Daily defined doses
DOT: Days of therapy

## References

[1] Cassini A, Hogberg LD, Plachouras D, Quattrocchi A, Hoxha A, Simonsen GS (2019). Attributable deaths and disability-adjusted life-years caused by infections with antibiotic-resistant bacteria in the EU and the European Economic Area in 2015: a population-level modelling analysis. Lancet Infect Dis.

[2] Chatterjee A, Modarai M, Naylor NR, Boyd SE, Atun R, Barlow J (2018). Quantifying drivers of antibiotic resistance in humans: a systematic review. Lancet Infect Dis.

[3] David S, Reuter S, Harris SR, Glasner C, Feltwell T, Argimon S (2019). Epidemic of carbapenem-resistant *Klebsiella pneumoniae* in Europe is driven by nosocomial spread. Nat Microbiol.

[4] Barlam TF, Cosgrove SE, Abbo LM, MacDougall C, Schuetz AN, Septimus EJ (2016). Implementing an antibiotic stewardship program: guidelines by the infectious diseases society of America and the society for healthcare epidemiology of America. Clin Infect Dis.

[5] Baur D, Gladstone BP, Burkert F, Carrara E, Foschi F, Dobele S (2017). Effect of antibiotic stewardship on the incidence of infection and colonisation with antibiotic-resistant bacteria and *Clostridium difficile* infection: a systematic review and meta-analysis. Lancet Infect Dis.

[6] Davey P, Marwick CA, Scott CL, Charani E, McNeil K, Brown E (2017). Interventions to improve antibiotic prescribing practices for hospital inpatients. Cochrane Database Syst Rev.

[7] Vincent JL, Sakr Y, Singer M, Martin-Loeches I, Machado FR, Marshall JC (2020). Prevalence and outcomes of infection among patients in intensive care units in 2017. JAMA.

[8] Timsit JF, Bassetti M, Cremer O, Daikos G, de Waele J, Kallil A (2019). Rationalizing antimicrobial therapy in the ICU: a narrative review. Intensive Care Med.

[9] De Waele JJ, Akova M, Antonelli M, Canton R, Carlet J, De Backer D (2018). Antimicrobial resistance and antibiotic stewardship programs in the ICU: insistence and persistence in the fight against resistance. A position statement from ESICM/ESCMID/WAAAR round table on multi-drug resistance. Intensive Care Med.

[10] Kollef MH, Bassetti M, Francois B, Burnham J, Dimopoulos G, Garnacho-Montero J (2017). The intensive care medicine research agenda on multidrug-resistant bacteria, antibiotics, and stewardship. Intensive Care Med.

[11] Roberts JA, Paul SK, Akova M, Bassetti M, De Waele JJ, Dimopoulos G (2014). DALI: defining antibiotic levels in intensive care unit patients: are current beta-lactam antibiotic doses sufficient for critically ill patients?. Clin Infect Dis.

[12] Roberts JA, Joynt G, Lee A, Choi G, Bellomo R, Kanji S (2020). The effect of renal replacement therapy and antibiotic dose on antibiotic concentrations in critically ill patients: data from the multinational SMARRT study. Clin Infect Dis.

[13] Wong G, Taccone F, Villois P, Scheetz MH, Rhodes NJ, Briscoe S (2020). beta-Lactam pharmacodynamics in Gram-negative bloodstream infections in the critically ill. J Antimicrob Chemother.

[14] Tabah A, De Waele J, Lipman J, Zahar JR, Cotta MO, Barton G (2015). The ADMIN-ICU survey: a survey on antimicrobial dosing and monitoring in ICUs. J Antimicrob Chemother.

[15] Kallen MC, Binda F, Ten Oever J, Tebano G, Pulcini C, Murri R (2019). Comparison of antimicrobial stewardship programmes in acute-care hospitals in four European countries: a cross-sectional survey. Int J Antimicrob Agents.

[16] United Nations Statistics Division. Statistical yearbook; 2019.

[17] Maechler F, Schwab F, Geffers C, Meyer E, Leistner R, Gastmeier P (2014). Antibiotic stewardship in Germany: a cross-sectional questionnaire survey of 355 intensive care units. Infection.

[18] Delannoy M, Agrinier N, Charmillon A, Degand N, Dellamonica J, Leone M (2019). Implementation of antibiotic stewardship programmes in French ICUs in 2018: a nationwide cross-sectional survey. J Antimicrob Chemother.

[19] Pentella M, Weinstein MP, Beekmann SE, Polgreen PM, Ellison RT (2020). Impact of changes in clinical microbiology laboratory location and ownership on the practice of infectious diseases. J Clin Microbiol.

[20] Schouten J, De Angelis G, De Waele JJ (2020). A microbiologist consultant should attend daily ICU rounds. Intensive Care Med.

[21] Morris AM, Bai A, Burry L, Dresser LD, Ferguson ND, Lapinsky SE (2019). Long-term effects of phased implementation of antimicrobial stewardship in academic ICUs: 2007–2015. Crit Care Med.

[22] Maraolo AE, Ong DSY, Cimen C, Howard P, Kofteridis DP, Schouten J (2019). Organization and training at national level of antimicrobial stewardship and infection control activities in Europe: an ESCMID cross-sectional survey. Eur J Clin Microbiol Infect Dis.

[23] Guilhaumou R, Benaboud S, Bennis Y, Dahyot-Fizelier C, Dailly E, Gandia P (2019). Optimization of the treatment with beta-lactam antibiotics in critically ill patients-guidelines from the French Society of Pharmacology and Therapeutics (Societe Francaise de Pharmacologie et Therapeutique-SFPT) and the French Society of Anaesthesia and Intensive Care Medicine (Societe Francaise d'Anesthesie et Reanimation-SFAR). Crit Care.

[24] Tabah A, Bassetti M, Kollef MH, Zahar JR, Paiva JA, Timsit JF (2020). Antimicrobial de-escalation in critically ill patients: a position statement from a task force of the European Society of Intensive Care Medicine (ESICM) and European Society of Clinical Microbiology and Infectious Diseases (ESCMID) Critically Ill Patients Study Group (ESGCIP). Intensive Care Med.

[25] Abdul-Aziz MH, Alffenaar JC, Bassetti M, Bracht H, Dimopoulos G, Marriott D (2020). Antimicrobial therapeutic drug monitoring in critically ill adult patients: a position paper. Intensive Care Med.

[26] Rybak MJ, Le J, Lodise TP, Levine DP, Bradley JS, Liu C (2020). Therapeutic monitoring of vancomycin for serious methicillin-resistant *Staphylococcus aureus* infections: a revised consensus guideline and review by the American Society of Health-System Pharmacists, the Infectious Diseases Society of America, the Pediatric Infectious Diseases Society, and the Society of Infectious Diseases Pharmacists. Am J Health Syst Pharm.

[27] Cotta MO, Dulhunty JM, Roberts JA, Myburgh J, Lipman J (2016). Should beta-lactam antibiotics be administered by continuous infusion in critically ill patients? A survey of Australia and New Zealand intensive care unit doctors and pharmacists. Int J Antimicrob Agents.

[28] Abdul-Aziz MH, Lipman J, Akova M, Bassetti M, De Waele JJ, Dimopoulos G (2016). Is prolonged infusion of piperacillin/tazobactam and meropenem in critically ill patients associated with improved pharmacokinetic/pharmacodynamic and patient outcomes? An observation from the defining antibiotic levels in Intensive care unit patients (DALI) cohort. J Antimicrob Chemother.

[29] Fleuren LM, Roggeveen LF, Guo T, Waldauf P, van der Voort PHJ, Bosman RJ (2019). Clinically relevant pharmacokinetic knowledge on antibiotic dosing among intensive care professionals is insufficient: a cross-sectional study. Crit Care.

[30] Dyar OJ, Huttner B, Schouten J, Pulcini C (2017). Esgap. What is antimicrobial stewardship?. Clin Microbiol Infect.

[31] De Waele JJ, Schouten J, Beovic B, Tabah A, Leone M (2020). Antimicrobial de-escalation as part of antimicrobial stewardship in intensive care: no simple answers to simple questions—a viewpoint of experts. Intensive Care Med.

