# Supplementary material for: Antimicrobial stewardship, therapeutic drug monitoring and infection management in the ICU: results from the international A- TEAMICU survey
Source: Ann Intensive Care. 2021 Aug 26;11:131. doi: 10.1186/s13613-021-00917-2 (PMC8390725; doi:10.1186/s13613-021-00917-2)
Supplement: Supplementary file 1 — Additional file 1. A-TEAM-ICU Questionnaire. [file 13613_2021_917_MOESM1_ESM.docx]

**A-TEAM-ICU Questionnaire**

**Part 1: Hospital Information**

1. **In which country are you employed?**

Answers available as a drop-down menu

2. **What is the hospital type?**

□ Academic hospital

□ Non-academic teaching hospital

□ General non-teaching hospital

3. **Please state the total number of ICU beds in your hospital**

Numbers to be entered as free text

4**. How long is your experience in intensive care medicine?**

□ < 2 years

□ 2-5 year

□ 5-10 years

□ 10-20 years

□ > 20 years

5. **Have you got specific training in antibiotic therapy or infection management?**

□ Yes

□ No

6. **Are you the most experienced or well-trained intensivist of your service for infection management?**

□ Yes

□ No

7. **Does your hospital employ an Infectious Diseases (ID) specialist?**

□ Yes

□ No

□ No, but can be consulted from an external location

□ I don´t know

8. **Does your hospital employ a Clinical Microbiologist?**

□ Yes

□ No

□ No, but can be consulted from an external location

□ I don´t know

9. **What type of Medical Record does your ICU use?**

□ On paper

□ Electronic

**Part 2: Organisation of an Antimicrobial Stewardship Program**

10. **Does your hospital have a formal Antimicrobial Stewardship Program (ASP) to ensure appropriate antimicrobial use?**

□ Yes

**Antimicrobial Stewardship Program (ASP)** = a coordinated hospital program that promotes the appropriate use of antimicrobials to reduce the development of antimicrobial resistance

□ No

□ I don´t know

11. **Is an Antimicrobial Stewardship team available in your Intensive Care Unit?**

□ Yes

**Antimicrobial Stewardship team (A-team) =** a multi-disciplinary team (>than one staff member), that monitors antibiotic use and supports clinical decisions to ensure appropriate antibiotic use

□ No

□ I don´t know

12. **If yes in question 11: which type of professionals are part of the A-team?** **(multiple answers possible)**

□ Infectious Diseases specialist

□ Clinical Microbiologist

□ Clinical Pharmacist

□ Pediatrician

□ Quality of care officer

□ Infection prevention specialist

□ Nurse Epidemiologist

□ Intensivist

□ Other type of care professional (free text option for this)

□ I don´t know

13. **If yes in question 11: How often do the whole A-Team or members of it come to your ICU?**

□ Daily

□ Several times a week

□ Weekly

□ Never

□ on demand

□ Other (free text)

14. **Does your hospital restrict the use of certain antimicrobials and require a formal authorization of their use?**

□ Yes

□ No

□ I don´t know

15. **If yes in question 14:** **How does your ICU implement the restricted use of selected antimicrobial agents? (multiple answers possible)**

□ Pre-authorization of restricted agents (e.g. mandatory external review before prescription of a restricted agent)

□ Post-authorization of restricted agents (e.g. mandatory external review shortly after prescription of a restricted agent)

□ Formulary restriction (e.g. restricted agents are not available in the hospital at all)

□ Antibiotic Order forms (e.g. mandatory documentation of clinical indication when prescribing restricted agents)

□ Computerized alert to a control authority when prescribing restricted agents

□ Automatic stop orders for restricted agents (e.g. restricted agents can only be prescribed for a short time and authorization must be sought for extension)

□ Mandatory bedside consultation by control authority (e.g. A-Team) when prescribing restricted agents

□ Telephone feedback by control authority after prescription of restricted agents

□ Post-prescription review (e.g. feedback and advice after prescribing restricted agents)

□ Restrictions are monitored continuously in all patients

□ Restrictions are monitored occasionally in all patients

□ Other (free text option for this)

□ I don´t know

16. **Does your hospital have local guidelines for the treatment of common infectious diseases?**

□ Yes

**Local guidelines** = facility-specific evidence based treatment recommendations that assist professionals in their decision making on diagnostics and treatment for a specific disease

□ No

□ I don´t know

17. **If yes in question 16:** **Are these local guidelines based on local antimicrobial susceptibility?**

□ Yes

□ No

□ I don´t know

18. **Does your ICU have standardized criteria for (multiple answers possible):**

□ Streamlining or de-escalation of empirical antimicrobial therapy

□ Dose optimization (e.g. Therapeutic Drug Monitoring)

□ Discontinuation of antimicrobial therapy (e.g. Procalcitonin-Algorithm)

□ Duration of therapy

□ Surgical prophylaxis

□ None of these

19. **Does your ICU have a written policy that requires prescribers to document the indication of antimicrobial prescriptions in the medical records (e.g. when starting a new antimicrobial)?**

□ Yes

□ No

□ I don´t know

20. **Does your ICU monitor the quantity of antimicrobial use?**

□ Yes

**Monitoring** = the continuous measurement and reporting of relevant aspects of clinical care to ensure that stewardship goals are met

□ No

□ I don´t know

21. **If yes in question 20: What statistical measure does your ICU apply for the quantitative reporting of antimicrobial use?**

□ Defined Daily Dose (DDD) [by grams]

□ Days of Therapy (DOT) [by counts]

□ Other (free text option for this)

□ I don´t know

22. **Does the microbiology department in your hospital produce cumulative antimicrobial susceptibility reports at least annually?**

□ Yes

□ No

□ I don´t know

23. **Does your hospital perform mandatory bedside consultations by infectious disease specialists for special types of infections in the ICU?**

□ Yes

□ No

□ I don´t know

24**. If yes in question 23: For which type of infection has your hospital agreed to perform these bedside ID-consultations in the ICU?**

□ Staphylococcus aureus bacteremia

□ Infective endocarditis (native valve, prosthetic valve, pacemaker)

□ Infection of a prosthetic joint

□ Infection of a vascular prosthesis

□ Invasive fungal infection/

□Other (free text option for this)

**Part 3: Therapeutic Drug Monitoring**

25. **Does your ICU have written guidelines for antibiotic dosing?**

□ Yes, a local guideline of the hospital/ICU

□ Yes, a national guideline

□ No

□ I don´t know

26. **Does your ICU use therapeutic drug monitoring (TDM) of antimicrobial drugs?**

□ Yes

□ No

□ I don´t know

27. **If yes in question 26: Which department performs the drug measurements of antimicrobials in your hospital?**

□ Clinical pharmacy

□ Clinical chemistry laboratory

□ Microbiology

□ other (free text option for this)

□ I don´t know

28. **If yes in question 26: Which department advises on the use of these measurements in clinical practice in your ICU?**

□ Clinical pharmacy

□ Clinical chemistry laboratory

□ Microbiology

□ Infectious Diseases specialist

□ Intensivist

□ other (free text option for this)

□ I don´t know

29. **If yes in question 26: For which antimicrobials does your ICU have TDM available? (multiple answers possible)**

□ Penicillins with or without ß-lactamase inhihitors (Oxacillin, Ampicillin/Sulbactam, Piperacillin/Tazobactam etc.)

□ Cephalosporins (Cefuroxim, Cefepime, Ceftazidime etc.)

□ Quinolones (Levofloxacin, Ciprofloxacin, etc.)

□ Glycopeptides (Vancomycin, Teicoplanin)

□ Aminoglycosides (Gentamicin, Tobramycin, etc.)

□ Carbapenems (Meropenem, Imipenem, Ertapenem, etc.)

□ Linezolid

□ Daptomycin

□ Azole antifungals (Fluconazole, Voriconazole, Posaconazole, etc.)

□ Echinocandine antifungals (Anidulafungin, Caspofungin, Micafungin)

□ Colistin

□Other (free text option for this)

30. **Does your ICU use prolonged/continuous infusion of any antimicrobial?**

□ Yes

□ No

□ I don´t know

31. **If yes in question 30: Which antimicrobials are applied via prolonged/continuous infusion in your ICU?**

□ Penicillins with or without ß-lactamase inhihitors (Oxacillin, Ampicillin/Sulbactam, Piperacillin/Tazobactam etc.)

□ Cephalosporins (Cefuroxim, Cefepime, Ceftazidime etc.)

□ Glycopeptides (Vancomycin, Teicoplanin)

□ Carbapenems (Meropenem, Imipenem, Ertapenem, etc.)

□ Linezolid

□ Azole antifungals (Fluconazole, Voriconazole, Posaconazole, etc.)

□Other (free text option for this)

32. **If yes in question 30: Is TDM available for every antimicrobial that is applied via prolonged/continuous infusion in your ICU?**

□ Yes

□ No

□ I don´t know

**Part 4: Education in Antimicrobial Stewardship**

33. **Does your ICU provide education to doctors on Antimicrobial Stewardship?**

□ Yes

□ No

□ I don´t know

34. **If yes in question 33: Is this type of education:**

□ Voluntary

□ Mandatory

35. **If yes in question 33: Which subjects are discussed in the training of doctors? (multiple answers possible)**

□ Antimicrobial resistance (local and/or general aspects)

□ Specific syndromes (e.g. *S. aureus*, pneumonia, endocarditis)

□ Use and supervision of restricted medication (e.g. pre-authorisation)

□ Switch from intravenous to oral route

□ Streamlining or de-escalation of antimicrobial therapy

□ Therapeutic drug monitoring (TDM)

□ Use and relevance of point prevalence surveys and audits

□ Other (free text option for this)

□ I don´t know

36. **Is there anything else you would like to share with us?**

Free text option for this
